# Mechanism of M2 macrophage-derived extracellular vesicles carrying lncRNA MEG3 in inflammatory responses in ulcerative colitis

**DOI:** 10.1080/21655979.2021.2010368

**Published:** 2021-12-11

**Authors:** Yu-Xuan Wang, Cheng Lin, Lu-Jia Cui, Tao-Zhi Deng, Qiu-Min Li, Feng-Ying Chen, Xin-Pu Miao

**Affiliations:** Department of Gastroenterology, Hainan General Hospital, Haikou, P.R. China

**Keywords:** Ulcerative colitis, inflammatory reaction, M2 macrophages, extracellular vesicles, LncRNA MEG3

## Abstract

Ulcerative colitis (UC) is a chronic inflammatory disease of the colon. M2 macrophages possess certain anti-inflammation activity. Accordingly, the current study set out to investigate the potential mechanism of M2 macrophage-derived extracellular vesicles (M2-EVs) in UC inflammation. Firstly, mouse peritoneal macrophages were induced to M2 phenotype, and M2-EVs were isolated. , the murine model of UC was established, and the length and weight of the colon, disease activity index (DAI), apoptosis, and inflammatory response of UC mice were measured. Young adult mouse colon (YAMC) cells were induced with the help of lipopolysaccharide. LncRNA maternally expressed 3 (LncRNA MEG3), miR-20b-5p, and cAMP responsive element binding protein 1 (CREB1) expression patterns were detected in UC models. In addition, we analyzed the binding relationship among MEG3, miR-20b-5p, and CREB1. UC mice presented with shortened colon length, lightened weight, increased DAI score, enhanced apoptosis, and significant inflammatory cell infiltration, while M2-EVs reversed these trends. *In vitro*, M2-EVs increased UC cell viability and reduced inflammation. Mechanistic experimentation revealed that M2-EVs transferred MEG3 into YAMC cells to up-regulate MEG3 expression and promote CREB1 transcription by competitively binding to miR-20b-5p. Moreover, up-regulation of MEG3 in M2-EVs enhanced the protective effect of M2-EVs on UC cells, while over-expression of miR-20b-5p attenuated the aforementioned protective effect of M2-EVs on UC mice and cells. Collectively, our findings revealed that M2-EVs carrying MEG3 enhanced UC cell viability and reduced inflammatory responses *via* the miR-20b-5p/CREB1 axis, thus alleviating UC inflammation.

## Introduction

Ulcerative colitis (UC) is a highly-prevalent, chronic, and idiopathic inflammatory disease of the colonic mucosa, beginning at the rectum and usually extending to the proximal part of the entire colon in a continuous manner [1]. Clinically, UC is typically manifested as abdominal pain, diarrhea, and bloody stools, all of which can dramatically worsen the quality of life of UC patients [2]. It is well established that a myriad of complex effects of environmental and host factors can augment the susceptibility of UC, whereas UC is often triggered by events that disturb the mucosal barrier, impair the healthy balance of intestinal microbiota, and aberrantly stimulate the intestinal immune response [3]. Unfortunately, due to the poor specificity and insufficient means to inhibit inflammation, the clinical efficacy of currently-available pharmacotherapy is not very ideal, as manifested by high recurrence rates and increased risk of carcinogenesis [4]. Against this backdrop, it would be prudent to expand the search for potent and feasible therapeutic targets to alleviate the inflammatory response for UC patients.

Macrophages are well-established as a heterogeneous group of immune cells implicated in chronic inflammatory and autoimmune disorders, such that macrophages also confer critical roles in UC pathogenesis by regulating the intestinal immune system and inflammatory response [5]. Macrophages can differentiate into the classically activated macrophages (M1) and the alternatively activated macrophages (M2); M1 macrophages bear pro-inflammatory and anti-microbial activities, whereas M2 macrophages are associated with anti-inflammatory cytokines and scavenging molecules [6]. M1 macrophages are often increased and M2 macrophages are typically decreased in the inflammatory colon tissues of UC patients, whereas the imbalance between M1 and M2 contributes to UC progression [7]. Interestingly, existing evidence suggests that administration of M2 macrophages into UC mice exerts a reducing effect on colon inflammation [8]. Therefore, searching novel therapies based on M2 macrophages could be a promising approach for UC treatment.

On a separate note, extracellular vesicles (EVs) are known as membrane-defined nanometer-sized vesicles released by cells, and have further garnered the attention of numerous researchers as a crucial carrier of intercellular communication [9]. Moreover, macrophage-derived EVs have emerged as critical mediators in the pathology of a plethora of inflammatory diseases [10]. Particularly, M2-EVs have been shown to play an effective anti-inflammatory role *via* the surface-bonded chemokine receptors and the anti-inflammatory cytokines released by M2 macrophages [11]. Mechanistically, EVs are capable of transferring proteins and genetic information (DNA, RNA, and primarily small non-coding RNAs) to participate in intercellular communication, while their lipid bilayer membrane effectively protects the cargoes from degradation during transportation [12]. Meanwhile, long non-coding RNAs (lncRNAs) are a group of transcripts longer than 200 nucleotides without an open reading frame [13]. Owing to the involvement of lncRNAs in the modulation of various biological processes including inflammation and immunity, it has been suggested that aberrant lncRNA expressions can interfere with the pathogenic mechanisms of UC [14]. One such lncRNA, namely lncRNA maternally expressed 3 (lncRNA MEG3), an imprinted lncRNA located at chromosome 14q32, has been previously identified to alleviate lipopolysaccharide (LPS)-induced intestinal injury in sepsis [15]. Furthermore, over-expression of lncRNA MEG3 can attenuate colonic ulcers in UC rats by augmenting the interleukin (IL)-10 expression [16]. Importantly, emerging evidences have revealed that MEG3 can be carried by EVs so as to regulate disease procession [17,18]. However, whether M2-EVs can influence the inflammatory response in UC by carrying lncRNA MEG3 remains unknown. Accordingly, we speculated that lncRNA MEG3 carried by M2-EVs may regulate the inflammatory response in UC through the miR-20b-5p/cAMP responsive element binding protein 1 (CREB1) axis. Thereafter, the current study set out to investigate the role of lncRNA MEG3 carried by M2-EVs in the inflammatory response in UC and its downstream molecular mechanism, in an effort to provide a novel theoretical basis for the treatment of UC.

## Materials and Methods

### Ethics statement

The current study was performed following the approval of the Ethical Committee of Hainan General Hospital. All animal experiments were implemented based on the *Guide for the Care and Use of Laboratory Animals* [19]. Extensive efforts were made to minimize the number and suffering of the experimental animals.

### Isolation and identification of M2 macrophages

Specific pathogen-free mice were procured from Vital River Laboratory Animal Technology Co., Ltd (Beijing, China) [SYXK (Beijing) 2017–0033]. Mouse M2 macrophages were isolated as described in previous literature [20]. Briefly, the mice were intraperitoneally administered with 5 mL pre-cooled serum-free RPMI-1640 medium, and the abdominal cavity was gently rubbed for 2–3 min. Subsequently, the peritoneal cavity was lifted with forceps after 5 min, and the peritoneal lavage fluid was extracted with a suction tube, and the peritoneal lavage repeated once. The lavage fluid was centrifuged at 4°C to discard the supernatant. Next, the obtained precipitated cells were suspended in RPMI-1640 medium comprising of 100 U/mL penicillin, 100 μg/mL streptomycin, and 10% fetal bovine serum (FBS). The cells were counted and seeded in 24-well plates (2 × 10^6^ cells/mL) and cultured at 37°C with 5% CO_2_ in air. The medium was renewed 24 h later. The adherent monolayer cells were collected as macrophages, and the non-adherent cells were discarded.

The collected macrophages were cultured in complete medium, and then M2 macrophages were obtained following a 24-h regimen of incubation with 20 ng/mL mouse interleukin-4 (IL-4) (Sigma-Aldrich, Merck KGaA, Darmstadt, Germany) [20]. Subsequently, the macrophages were collected, and cell morphology was observed under a microscope. For flow cytometry, the sample suspension (1 × 10^6^) was supplemented with 2 μL fluorescent antibodies [CD68 (ab53444, Abcam, Cambridge, MA, USA) and CD163 (ab182422, Abcam)] and homologous control on ice for 30 min. Next, the sample was rinsed with fluorescence-activated cell sorting buffer and fixed with 10% formalin. , flow cytometry was carried out for the detection of the positive rate of antigens.

### Treatment of M2 macrophages

The construction of the MEG3 over-expression lentivirus vector was entrusted to GenePharma (Shanghai, China). The vector containing the negative sequence that did not target any gene was adopted as the negative control (NC). Subsequently, these lentivirus vectors were packaged in 293 T cells (American Type Culture Collection, ATCC, Manassas, Virginia, USA) for 72 h, followed by virus particle collection. M2 macrophages were then infected with virus particles and 8 μg/mL Polybrene (Sigma-Aldrich) [21], and selected using puromycin (Sigma-Aldrich) for 7 days. Thereafter, the EVs (M2-EVs-LV-NC and M2-EVs-LV-MEG3) were isolated. Additionally, the presence of MEG3 in M2-EVs was identified using RNase A (Sigma-Aldrich, 2 mg/mL) treatment alone or co-treatment with Triton X-100 (0.1%).

### Isolation and identification of M2-EVs

EVs derived from M2 macrophages were isolated as described in previous literature [20]. Briefly, M2 macrophages were cultured in RPMI-1640 medium containing 10% EV-depleted FBS (EVs were depleted by 18-h centrifugation at 200,000 g) for 48 h. The medium was centrifuged at 2000 g and 4°C for 20 min to remove the debris and dead cells, and then centrifuged at 100,000 g and 4°C for 90 min to collect the particles. Following a phosphate-buffered saline (PBS) rinse, the EV-containing particles were subjected to ultracentrifugation again at 100,000 g and 4°C for 90 min and resuspended in PBS. The conditioned medium obtained by the addition of GW4869 (EV inhibitor, 20 μg/mL; Sigma-Aldrich) to macrophages was regarded as the GW group. Subsequently, the protein content of EVs was analyzed using bicinchoninic acid kits. The samples were dried at room temperature and observed under an 80 keV transmission electron microscope (TEM) (Hitachi, Tokyo, Japan). The Nanoparticle tracking analyzer (ZetaView) was adopted to measure the size and concentration of EVs. The specific markers of EVs such as CD63, CD81, and Calnexin were determined by means of Western blot assay.

### Establishment of the murine model of UC

Male C57BL/6 mice (aged 6–8 weeks; weighing 18–22 g) were obtained from Vital River Laboratory Animal Technology Co., Ltd, and maintained at 22 ± 2°C and relative humidity of 55 ± 5% and under 12 h light/dark cycles. All mice had *ad libitum* access to water and food. In order to establish the dextran sodium sulfate (DSS)-induced colitis model, the mice were administered water containing 5% DSS for 7 days, and then distilled water for 3 days [22]. Meanwhile, mice in the sham group were given distilled water for 10 days. For the detection of the effect of EVs on inflammatory responses, the mice were injected with M2-EVs (50 μg/mouse, injected every 2 days) or the same amounts of conditioned medium of the GW group *via* tail vein 1 day before DSS treatment [23]. In addition, by referring to previous literature [22,24], on the 4^th^ day, the mice were injected with miR-20b-5p agomir and control agomir every 2 days (100 μL; 3 mg/kg; GenePharma) via tail vein. On the 11^th^ day, all mice were sacrificed by means of an intraperitoneal injection of 100 mg/kg pentobarbital sodium. Subsequently, the colons were removed, and the entire length and weight of each colon were measured. One part of the removed colon was used for histopathological analysis, and the other part was homogenized with the Pro-prep^TM^ buffer for subsequent analyses.

### Evaluation of disease activity index (DAI)

During the experiment, the body weight, fecal consistency, and total blood in the feces were recorded daily to calculate the DAI scores [25]. Briefly, DAI was scored based on the following parameters: a) weight loss (0, no loss; 1, 1–5% loss; 2, 6–10% loss; 3, 10–25% loss; 4, over 15% loss); b) diarrhea (0, normal; 2, loose stool; 4, liquid stool); c) hematochezia (0, no bleeding; 2, slight bleeding; 4, gross bleeding). DAI = (score of weight loss + stool consistency + degree of hematochezia)/3.

### Hematoxylin and eosin (HE) staining

Colonic tissues were rinsed with cold PBS, fixed in 4% paraformaldehyde, dehydrated, paraffin-embedded, and sliced into 5 μm sections. Next, the sections were stained with hematoxylin and eosin (Sigma-Aldrich) [26]. The severity of colitis was subsequently determined by experienced pathologists using a double-blind method. The scoring criteria was as follows: score of 0, normal submucosa, mucosa, serosa, and colonic muscularis propria, no inflammatory cells; score of 1, inflammatory cells can be observed in submucosa and mucosa; score of 2, transmural inflammation; score of 3, small gastric ulcer with acute inflammation; score of 4, multiple large ulcers with transmural inflammation; score of 5, extensive ulcer with mucosal wall necrosis, an irregular villous mucosal surface, and transmural inflammation [27].

### Terminal deoxynucleotidyl transferase (TdT)-mediated dUTP nick end labeling (TUNEL) staining

Colonic tissues were fixed with 4% paraformaldehyde, dehydrated with gradient ethanol, embedded in paraffin, and sliced into 5 μm sections. Subsequently, the sections were stained with TUNEL kits (Roche, Penzberg, Germany) to evaluate the apoptosis in colon tissues [22], followed by observation under a light microscope (Olympus BH-2; Olympus, Tokyo, Japan).

### Cell culture and treatment

Conditionally immortalized murine colon epithelial cell line was procured from Crisprbio (#CE19668; Beijing, China), and then cultured in RPMI-1640 medium comprising of penicillin-streptomycin-glutamine and 5% FBS at 37°C with 5% CO_2_ in air [22]. Young adult mouse colon (YAMC) cells were treated with 1 μg/mL LPS for 24 h to simulate the inflammatory environment of UC *in vitro* [28]. After 30 min of pre-stimulation, YAMC cells were subjected to treatment with 100 μg/mL M2-EVs, with the conditioned medium of the GW group serving as the control. miR-20b-5p mimic and its NC were provided by GenePharma and transfected into YAMC cells using the Lipofectamine 2000 reagent (Invitrogen Inc., Carlsbad, CA, USA).

### Co-culture of Cy3-labeled and MEG3-transfected M2 macrophages with YAMC cells

M2 macrophages were treated with 0.25% trypsin and resuspended in RPMI 1640 medium containing 10% FBS (1 × 10^6^ cells/well). Subsequently, Cy3-labeled MEG3 (MEG3-Cy3; GenePharma) was transfected into the macrophages using Lipofectamine 2000 (Invitrogen). Next, the macrophages expressing MEG3-Cy3 were seeded into 6-well plates and co-cultured with green fluorescent protein (GFP)-transfected YAMC cells in the Transwell chamber for 2–4 days [29]. The cells were observed by means of confocal microscopy (Leica Microsystems, Mannheim, Germany).

### Cell counting kit-8 (CCK-8) assay

Cell viability was measured with the help of CCK-8 assay kits [25]. Briefly, 100 μL cell suspension (10^4^ cells/mL) was cultured with 10 μL CCK-8 reagent (Beyotime, Shanghai, China) for 4 h. Subsequently, the absorbance at a wavelength of 450 nm was determined using a microplate reader (Bio-Rad, Hercules, CA, USA).

### Flow cytometry

After treatment, the cells were rinsed with PBS twice. Next, 1 × 10^5^ cells were incubated with Annexin V-FITC and propidium iodide (PI) provided with the Annexin V-FITC/PI apoptosis detection kits (Sigma-Aldrich) at room temperature. Briefly, the cells were suspended in 100 μL Annexin buffer solution, and the suspension was added with 5 μL Annexin V and 1 μL PI and incubated in conditions void of light for 15 min. The stained cells were analyzed using a flow cytometer (BD Biosciences, San Jose, CA, USA) [30].

### Nuclear/cytosol fractionation assay

Nuclear and cytosolic fractions were obtained with the help of nuclear and cytoplasmic protein extraction kits (Beyotime) [23]. Briefly, 1 × 10^7^ cells were rinsed with PBS, collected using 200 μL cytoplasmic protein extraction reagent A, and then incubated with 10 μL cytoplasmic protein extraction reagent B on ice. Subsequently, the supernatant was centrifuged at 12,000 g and 4°C for 10 min to separate the nuclei from the cytoplasm. After separation, the nuclei were resuspended in 50 μL nuclear protein extraction buffer and agitated on ice for 30 min. Following centrifugation at 12,000 g and 4°C for 10 min, the supernatant was collected as the nuclear extract for subsequent analyses.

### RNA fluorescence in situ hybridization (FISH) assay

Cy3-labeled MEG3 probe was procured from GenePharma. YAMC cells were fixed with 4% paraformaldehyde for 10 min, treated with 0.1% Triton X-100 for 5 min, and then subjected to hybridization with the FISH probe at 37°C overnight [31]. The following day, the nuclei were stained with 4ʹ,6-diamidino-2-phenylindole for 8 min. Nuclei images were obtained using a confocal microscope.

### RNA immunoprecipitation (RIP)

As described in previous literature [32], Magna RIP kits (Sigma-Aldrich) were adopted to detect the binding between MEG3, miR-20b-5p, and argonaute2 antibody (Anti-Ago2) protein. Briefly, YAMC cells were lysed, and the supernatant was collected by means of centrifugation. A portion of the supernatant was used as the Input and did not participate in the magnetic bead incubation. The remaining portion of the supernatant was incubated with Anti-Ago2 (ab186733, Abcam) or IgG (ab172730, Abcam)-conjugated magnetic beads at 4°C overnight. The following day, reverse transcription quantitative polymerase chain reaction (RT-qPCR) was performed to detect the enrichment of MEG3 and miR-20b-5p.

### Dual-luciferase assay

MEG3 sequence or CREB1 3ʹ-UTR containing miR-20b-5p binding site was inserted into the pMIR-REPORT plasmid (Thermo Fisher Scientific, Waltham, MA, USA) to construct the wild-type plasmids of MEG3 and CREB1 (MEG3 WT and CREB1 WT), respectively. Subsequently, the MEG3 fragment of the mutant sequence or CREB1 3ʹ-UTR was inserted into the plasmid respectively to construct the mutant-type plasmids (MEG3 MUT and CREB1 MUT). The constructed luciferase reporter plasmids were then co-transfected with miR-20b-5p mimic or its NC (GenePharma) into 293 T cells (ATCC) using the Lipofectamine 2000 reagent (Invitrogen). After 48 h, a luciferase assay system (Promega, Madison, WI, USA) was employed for luciferase activity analysis [24].

### Enzyme-linked immunosorbent assay (ELISA)

Colonic tissue homogenate was prepared using a radio-immunoprecipitation assay (RIPA) buffer and centrifuged at 12,000 g and 4°C for 10 min to obtain the supernatant. Next, the cells were centrifuged at 1,000 g for 10 min to collect the supernatant. The concentration of tumor necrosis factor-α (TNF-α) (ab208348, Abcam), IL-1β (ab197742, Abcam), monocyte chemoattractant protein-1 (MCP-1) (ab208979, Abcam), and IL-10 (ab255729, Abcam) were subsequently determined using ELISA kits. Furthermore, the absorbance at a wavelength of 450 nm was determined using a microplate reader (Bio-Rad) [25].

### RT-qPCR

Total RNA content was extracted from samples using the TRIzol reagent (Invitrogen) [24], and the concentration of RNA was determined with the NanoDrop 2000 instrument (Thermo Fisher Scientific). Subsequently, the extracted RNA was reverse-transcribed into cDNA using ReverTra Ace qPCR RT kits (Toyobo, Osaka, Japan). The SYBR Master Mix (Promega) and CDX 96 RealTime PCR System were adopted for PCR. The primers are shown in Table 1. The relative expression of genes was calculated using the 2^−ΔΔCt^ method, with glyceraldehyde-3-phosphate dehydrogenase (GAPDH) or U6 serving as the internal reference [33].

**Table 1.** Primer sequence for RT-qPCR

Name	Sequence (5ʹ-3ʹ)
MEG3	F: ATGAGAGAGAGAACAGCGAGAA
	R: GTCTATGGACTGAAGATGTGAC
miR-20b-5p	F: GCCGAGCAAAGTGCTCATAGT
	R: CTCAACTGGTGTCGTGGA
CREB1	F: ATGCCAGCAGCTCATGCAACAT
	R: GATTTGTGGCAGTAAAGGTCC
CCL22	F: ATGGCTACCCTGCGTGTCCCA
	R: CTAGGACAGTTTATGGAGTAGC
PPARγ	F: ATGGTTGACACAGAGATGCC
	R: TCCTTGTAGATCTCCTGGAGCA
Bcl-2	F: ATGGCGCAAGCCGGGAGAAC
	R: TCACTTGTGGCCCAGGTATGCA
Bax	F: TGAACAGATCATGAAGACAGG
	R: TCAGCCCATCTTCTTCCAGATG
U6	F: GCTCGCTTCGGCAGCACATATA
	R: GGAACGCTTCACGAATTTGCG
GAPDH	F: ATGCTGCCCTTACCCCGGGGT
	R: TTACTCCTTGGAGGCCATGTAG

**Note**: RT-qPCR: reverse transcription quantitative polymerase chain reaction; MEG3: maternally expressed 3; miR-20b-5p: micro-20b-5p; CREB1: cAMP responsive element binding protein 1; CCL22: chemokine (C-C motif) ligand 22; PPARγ: peroxisome proliferator activated receptor gamma; Bcl-2: B-cell lymphoma protein 2; Bax: BCL2-associated X protein; GAPDH: glyceraldehyde-3-phosphate dehydrogenase

### Western blot assay

Total protein content was extracted from EVs and its control using RIPA buffer containing 1 mM phenylmethylsulfonyl fluoride (Solarbio, Beijing, China) [24]. Equal amounts of protein were separated by means of 10% sodium dodecyl sulfate-polyacrylamide gel electrophoresis, and then transferred onto polyvinylidene fluoride membranes. Subsequently, the membranes were incubated with CD63 (ab217345, dilution ratio of 1:1000, Abcam), CD9 (ab92726, dilution ratio of 1:2000, Abcam), and Calnexin (ab133615, dilution ratio of 1:1000, Abcam) at 4°C overnight. The following day, the membranes were washed with TBST (Solarbio) 3 times and incubated with the secondary antibody (ab205718, dilution ratio of 1:2000, Abcam) for 2 h. The NIH Image J software (National Institutes of Health, Bethesda, Maryland, USA) was adopted for analyses of gray values.

### Bioinformatics analysis

The subcellular localization of MEG3 was predicted through the online LncLocator database (http://www.csbio.sjtu.edu.cn/bioinf/lncLocator/) [34]. In addition, the downstream miRNAs of MEG3 were predicted through the DIANA tools (http://carolina.imis.athena-innovation.gr/diana_tools/web/index.php) [35] and RNAInter database (http://www.rna-society.org/rnainter/) [36]. Moreover, the downstream target genes of miR-20b-5p were predicted through the Starbase (http://www.targetscan.org/vert_71/) [37] and TargetScan databases (http://starbase.sysu.edu.cn/index.php) [38]. The binding site between miR-20b-5p and MEG3 was then analyzed with the RNA22 v2 database (https://cm.jefferson.edu/rna22/Interactive/) [39], and the binding site between miR-20b-5p and CREB1 was analyzed using the TargetScan database.

## Statistical analysis

Data analyses and map plotting were performed using the SPSS 21.0 statistical software (IBM Corp., Armonk, NY, USA) and GraphPad Prism 8.0 (GraphPad Software Inc., San Diego, CA, USA). Measurement data complied with the assumption of normality and homogeneity of variance. Measurement data are expressed as mean ± standard deviation. One-way or two-way analysis of variance (ANOVA) was adopted for comparisons among multiple groups, following Tukey’s multiple comparison test or Sidak’s multiple comparison test. A value of *p* < 0.01 was regarded statistically significant.

## Results

The current study set out to explore the effect and mechanism of M2-EVs carrying lncRNA MEG3 on the inflammatory responses of UC. Firstly, we explored effects of M2-EVs on inflammatory response of UC mice and LPS-induced YAMC cells. In addition, we also investigated the molecular mechanism of lncRNA MEG3 carried by M2-EVs in UC inflammatory responses *in vivo* and *in vitro*. The obtained findings confirmed that M2-EVs could reduce the inflammatory response of UC mice and LPS-induced YAMC cells. Mechanistical investigation further revealed that M2-EVs up-regulated the expression of MEG3 in cells by delivering MEG3 into colonic epithelial cells, and then promoted the transcription of CREB1 by competitive-binding to miR-20b-5p. Furthermore, we confirmed that overexpression of miR-20b-5p *in vivo* and *in vitro* reduced the protective effect of M2-EVs on UC inflammatory responses by diminishing the transcription of CREB1.

### Isolation of M2 macrophages and M2-EVs

**Figure 1.**
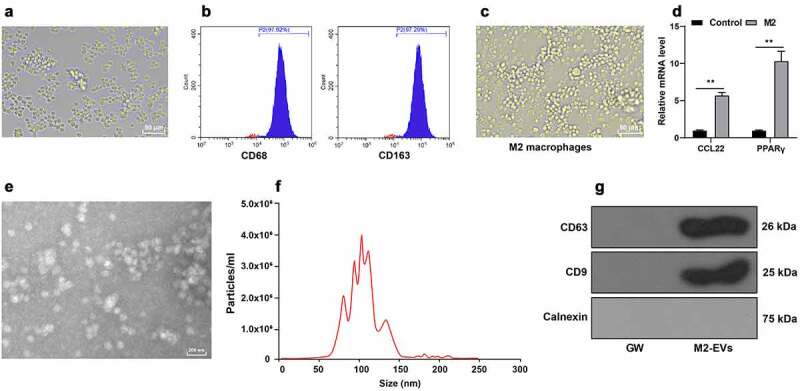
Identification of M2 macrophages and M2-EVs. Mouse peritoneal macrophages were isolated. a: The morphology of macrophages was observed under microscope. b: The markers of M2 macrophages (CD68 and CD163) were analyzed using flow cytometry after IL-4 induction. c: The morphology of M2 macrophages was observed under the microscope. d: The polarization molecules of M2 macrophages (CCL22 and PPARγ) were determined using RT-qPCR. e: The morphology of M2-EVs was observed under transmission electron microscope. f: The size and concentration of M2-EVs were measured using Nanoparticle tracking analyzer. g: The specific markers of M2-EVs (CD63, CD81, and Calnexin) were determined using Western blot. The experiment was repeated 3 times independently. Data are presented as mean ± standard deviation. Data in panel D were analyzed using two-way ANOVA, followed by Sidak’s multiple comparison test, **p < 0.01

The hard-done work of our peers has indicated that M2-EVs can alleviate inflammation [11,20], however, the effects of M2-EVs on the inflammatory response in UC remain elusive. Accordingly, we isolated mouse peritoneal macrophages and observation under a microscope illustrated that the obtained cells conformed to the morphological characteristics of macrophages (Figure 1(a)). In addition, the results of flow cytometry exhibited that the cells were positive for CD68 and CD163 (Figure 1(b)). Subsequently, we adopted IL-4 to induce M2 polarization. Microscopic observation demonstrated the cell morphology was obviously increased, mainly round cells (Figure 1(c)). Thereafter, we detected the polarization molecules of M2 macrophages including chemokine (C-C motif) ligand 22 (CCL22) and peroxisome proliferator activated receptor gamma (PPARγ), and found that CCL22 and PPARγ mRNA levels were both notably increased following IL-4 induction (*p* < 0.01, Figure 1(d)). After isolation, M2-EVs were observed under an electron microscope, which revealed that M2-EVs presented with a bilayer structure (Figure 1(e)), with an average diameter of 100 nm and a concentration of 4.0 × 10^6^ particles/mL (figure 1(f)). Moreover, we also observed that M2-EVs expressed CD9 and CD63, but not Calnexin (Figure 1(g)). Together, these findings validated the successful isolation of M2 macrophages and M2-EVs.

### M2-EVs reduced inflammatory response in UC mice

**Figure 2.**
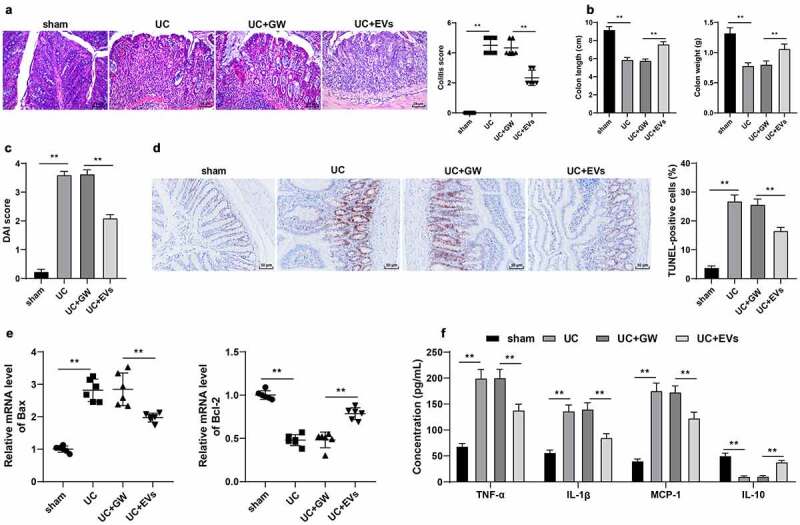
M2-EVs reduced the inflammatory response in UC mice. The murine model of UC was established by DSS induction. M2-EV or GW4869-treated conditioned medium was injected into UC mice, and the colon tissues were collected on the 11^th^ day. a: HE staining and UC score. b: Colon length and weight of mice in each group. c: DAI score of mice in each group. d: Cell apoptosis in colon tissues was measured using TUNEL staining. e: The levels of apoptosis-related factors (Bax and Bcl-2) were determined using RT-qPCR. f: The contents of inflammatory cytokines (TNF-α, IL-1β, MCP-1, and IL-10) were determined using ELISA. N = 6. Data in panels B/C/D/F are presented as mean ± standard deviation. Data in panels A-E were analyzed using one-way ANOVA, and data in panel F were analyzed using two-way ANOVA, followed by Tukey’s multiple comparisons test, **p < 0.01

To explore the effect of M2-EVs on the inflammatory response of UC mice, we adopted DSS to establish a murine models of UC. Subsequent results of HE staining illustrated that the colonic mucosa of UC mice was seriously damaged with inflammatory cell infiltration, and the colitis score was significantly elevated; meanwhile, there was a reduction in colon injury after M2-EVs treatment (*p* < 0.01, Figure 2(a)). In addition, UC mice presented with shortened length and lesser weight of the colon, and increased DAI score, while treatment with M2-EVs notably reversed the above trends (*p* < 0.01, Figure 2(b-c)). Furthermore, TUNEL staining exhibited that the apoptosis rate was increased after DSS treatment, while M2-EVs treatment brought about a reduction in the apoptosis rate (*p* < 0.01, Figure 2(d)). Additionally, we observed that DSS up-regulated BCL2-associated X protein (Bax) mRNA levels and down-regulated B-cell lymphoma protein 2 (Bcl-2) mRNA levels, while M2-EVs treatment resulted in opposing trends (*p* < 0.01, Figure 2(e)). Meanwhile, the results of ELISA demonstrated that DSS treatment increased the contents of pro-inflammatory factors (TNF-α, IL-1β, and MCP-1), and decreased the content of anti-inflammatory factor (IL-10), whereas M2-EVs significantly reduced the inflammatory response in UC mice (*p* < 0.01, figure 2(f)). Altogether, these findings indicated that M2-EVs reduced the inflammatory response and apoptosis in UC mice.

### M2-EVs attenuated LPS-induced cellular inflammation

**Figure 3.**
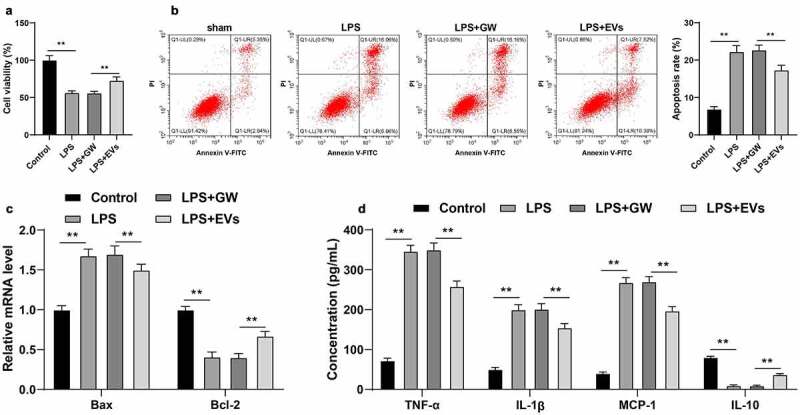
M2-EVs alleviated LPS-induced cellular inflammation. YAMC cells were treated with LPS to simulate the inflammatory environment of UC *in vitro*, and then treated with M2-EVs or GW4869-treated conditioned medium. a-b: Cell viability and apoptosis were measured using CCK-8 assay (a) and flow cytometry (b). c: Bax and Bcl-2 mRNA level were determined using RT-qPCR. d: The contents of inflammatory cytokines (TNF-α, IL-1β, MCP-1, and IL-10) were determined using ELISA. The experiment was repeated 3 times independently. Data are presented as mean ± standard deviation. Data in panels A/B were analyzed using one-way ANOVA, and data in panels C/D were analyzed using two-way ANOVA, followed by Tukey’s multiple comparisons test, **p < 0.01

Furthermore, we verified the effect of M2-EVs on UC inflammation *in vitro*. Briefly, YAMC cells were treated with LPS to simulate the inflammatory environment of UC *in vitro*. Subsequent findings revealed that LPS treatment notably decreased the cell viability and increased apoptosis, while treatment with M2-EVs brought about the opposite results (*p* < 0.01, Figure 3(a-c)). In addition, we found that LPS treatment also increased the contents of TNF-α, IL-1β, and MCP-1, and decreased the content of IL-10, whereas M2-EV treatment reduced the levels of LPS-induced cell inflammation (*p* < 0.01, Figure 3(d)). Collectively, these findings validated that M2-EVs attenuated LPS-induced cell inflammation *in vitro*.

### M2-EVs carried MEG3 into cells

**Figure 4.**
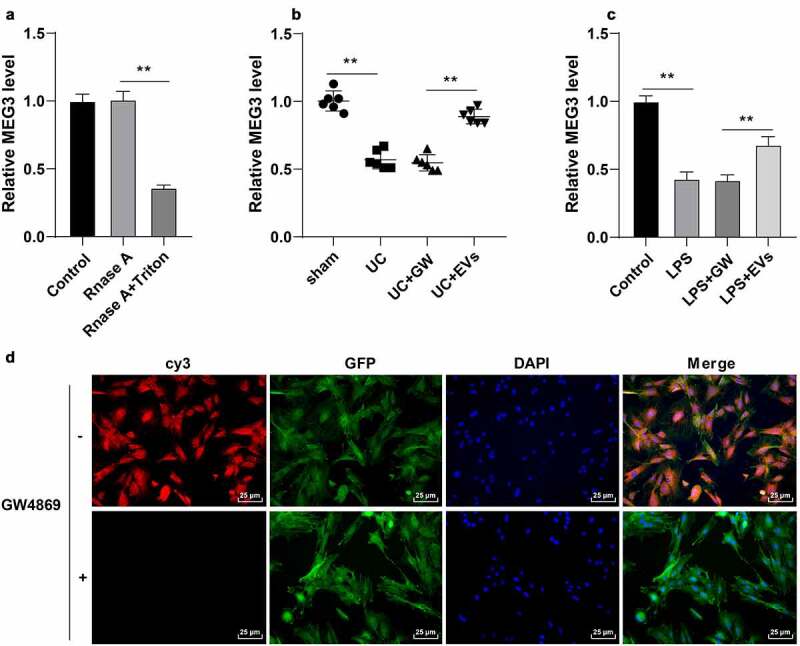
M2-EVs carried MEG3 into cells. A: MEG3 expression in M2 macrophages after Rnase A or Triton X-100 treatment was detected using RT-qPCR. B-C: MEG3 expression in tissues and cells was detected using RT-qPCR. D: M2 macrophages were transfected with Cy3 (red)-labeled meg3 and co-cultured with GFP (green)-transfected YAMC. N = 6. The experiment was repeated 3 times independently. Data in panels A/C are presented as mean ± standard deviation. Data in panels A-C were analyzed using one-way ANOVA, followed by Tukey’s multiple comparisons test, **p < 0.01

EVs are well-known to participate in intercellular communication by transferring large amounts of important substances such as lncRNAs [40]. Moreover, existing evidence suggests that lncRNA MEG3 can be carried by EVs, while MEG3 is poorly-expressed in LPS-induced intestinal injury in sepsis [15,41]. Herein, our findings illustrated that MEG3 expression in M2 macrophages was not influenced by RNase A treatment alone, while RNase A combined with Triton X-100 could decrease the MEG3 expression (*p* < 0.01, Figure 4(a)), suggesting that MEG3 was packaged in membrane structure and existed in M2-EVs. Furthermore, we observed that MEG3 expression levels were reduced in the *in vitro* and *in vivo* models of UC, while M2-EV treatment brought about increased MEG3 expressions (*p* < 0.01, Figure 4(b-c)). Furthermore, we transfected M2 macrophages with Cy3 (red)-labeled MEG3 and co-cultured with GFP (green)-transfected YAMC cells to verify whether M2-EVs could transfer MEG3 to YAMC cells. Subsequent findings illustrated a large amount of red fluorescence in the green fluorescence-labeled YAMC cells, while there was no red fluorescence in the above co-culture system after the addition of GW4869 (Figure 4(d)). Overall, these findings suggested that M2-EVs delivered MEG3 into YAMC cells to up-regulate MEG3 expression.

### M2-EVs protected UC cells by carrying MEG3

**Figure 5.**
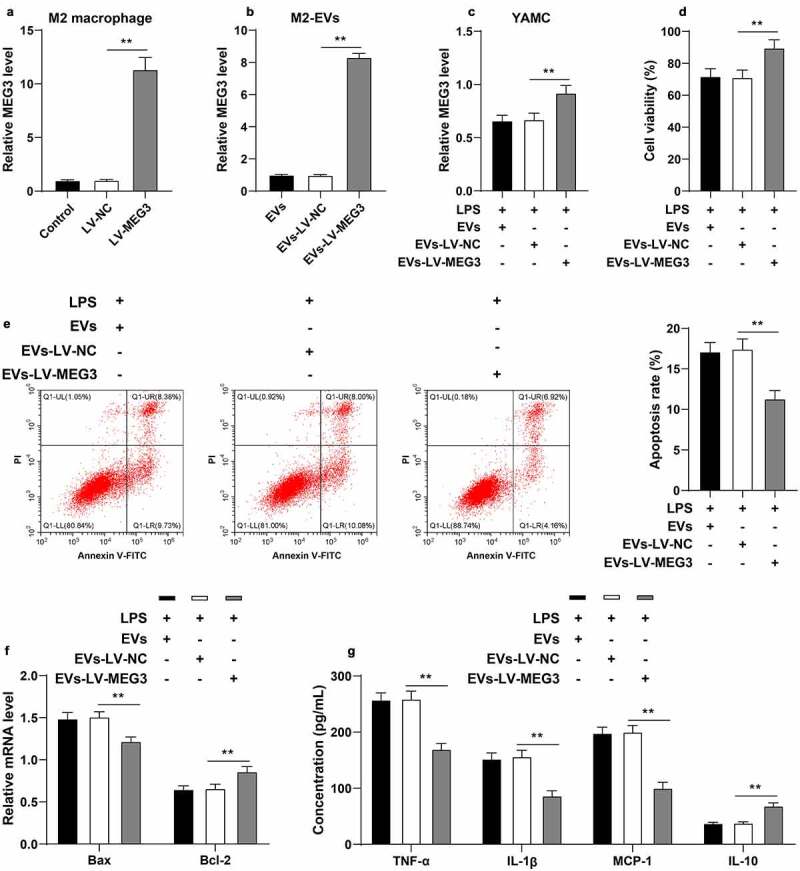
M2-EVs protected UC cells by carrying MEG3. M2 macrophages were transfected with LV-MEG3 or LV-NC, and then M2-EVs were isolated. a: MEG3 expression in M2 macrophages was detected using RT-qPCR. b: MEG3 expression in M2-EVs was detected using RT-qPCR. LPS-induced YAMC cells were treated with the above M2-EVs. c: MEG3 expression in YAMC cells was detected using RT-qPCR. d-e: Cell viability and apoptosis were measured using CCK-8 assay (d) and flow cytometry (e). f: Bax and Bcl-2 mRNA levels were determined using RT-qPCR. G: The contents of inflammatory cytokines (TNF-α, IL-1β, MCP-1, and IL-10) were determined using ELISA. The experiment was repeated 3 times independently. Data are presented as mean ± standard deviation. Data in panels A-E were analyzed using one-way ANOVA, and data in panels F/G were analyzed using two-way ANOVA, followed by Tukey’s multiple comparisons test, **p < 0.01

Furthermore, we verified the effect of MEG3 on UC inflammation with a series of experiments. Briefly, M2 macrophages were transfected with LV-MEG3 to up-regulate MEG3 expression in cells (*p* < 0.01, Figure 5(a)). EVs were isolated from LV-MEG3-transfected M2 macrophages (EVs-LV-MEG3), and subsequent observation revealed that MEG3 expression levels were increased in EVs-LV-MEG3 (*p* < 0.01, Figure 5(b)). In addition, we treated LPS-induced YAMC cells with M2-EVs-LV-MEG3, and found that MEG3 expression levels were increased in YAMC cells (*p* < 0.01, Figure 5(c)). After M2-EVs-LV-MEG3 treatment, LPS-induced YAMC cells presented with enhanced cell viability, decreased apoptosis (*p* < 0.01, Figure 5(d-f)), and reduced inflammation (*p* < 0.01, Figure 5(g)). Together, these findings indicated that M2-EVs delivered MEG3 into YAMC cells to up-regulate MEG3 expression in cells, thus conferring a protective role in UC cells.

### M2-EV-carried MEG3 competitively bound to miR-20b-5p to promote CREB1 transcription

**Figure 6.**
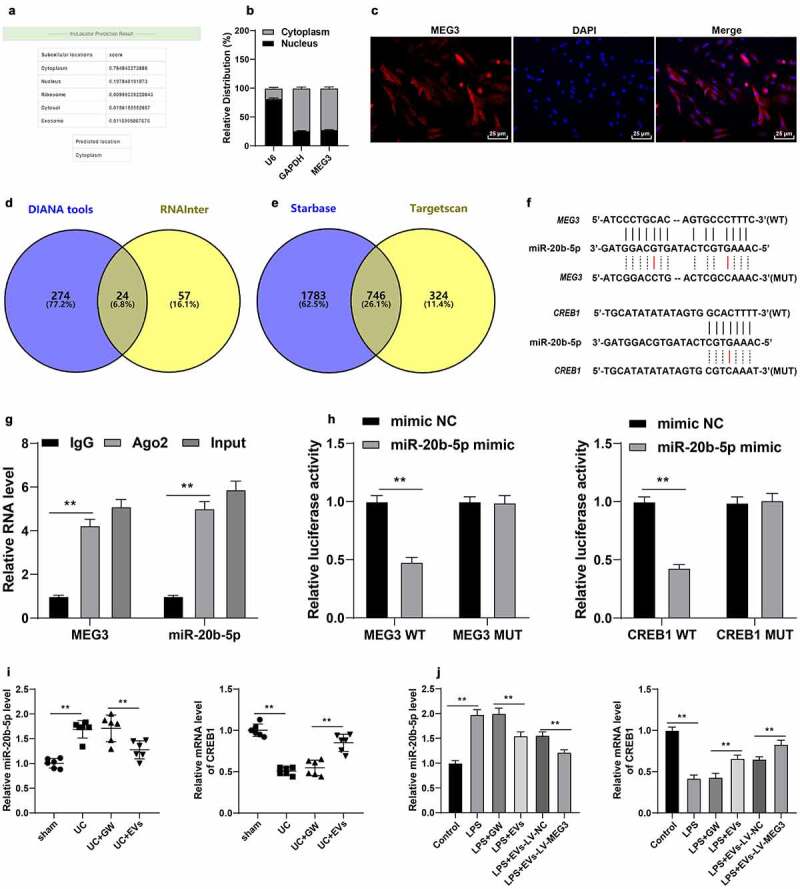
M2-EV-carried MEG3 competitively bound to miR-20b-5p to promote CREB1 transcription. a: The cellular localization of MEG3 was predicted through the LncLocator database (http://www.csbio.sjtu.edu.cn/bioinf/lncLocator/). b-c: The location of MEG3 in YAMC cells was confirmed using nuclear/cytosol fractionation assay (b) and RNA FISH (c). d: The downstream miRNAs of MEG3 were predicted through the DIANA tools (http://carolina.imis.athena-innovation.gr/diana_tools/web/index.php) and RNAInter (http://www.rna-society.org/rnainter/). e: The downstream mRNAs of miR-20b-5p were predicted through the Starbase (http://www.targetscan.org/vert_71/) and TargetScan (http://starbase.sysu.edu.cn/index.php). g-h: The binding relationships between miR-20b-5p and MEG3, and miR-20b-5p and CREB1 were analyzed using RIP (g) and dual-luciferase assay (h). i-j: miR-20b-5p and CREB1 expression in cells and tissues were detected using RT-qPCR. N = 6. The experiment was repeated 3 times independently. Data in panels B/G/H/J are presented as mean ± standard deviation. Data in panels I/J were analyzed using one-way ANOVA, and data in panels G/H were analyzed using two-way ANOVA, followed by Tukey’s multiple comparisons test or Sidak’s multiple comparisons test, **p < 0.01

To further explore the downstream mechanism of MEG3, we predicted the cellular localization of MEG3 using the LncLocator database (http://www.csbio.sjtu.edu.cn/bioinf/lncLocator/), which revealed that MEG3 was primarily localized in the cytoplasm (Figure 6(a)). Subsequent findings from the Nuclear/cytosol fractionation assay and RNA FISH assay confirmed that MEG3 was primarily located in the cytoplasm of YAMC cells (Figure 6(b-c)). Interestingly, lncRNAs located in the cytoplasm are known to participate in the pathogenesis of diseases by competitively binding to miRNAs [22]. Accordingly, we predicted the downstream miRNAs of MEG3 and obtained 24 candidate miRNAs (Figure 6(d)), among which miR-20b-5p was highly-expressed in UC [42]. The downstream mRNAs of miR-20b-5p were further predicted and intersected (Figure 6(e)), wherein CREB1 was found to be poorly-expressed in colitis [43]. RIP and dual-luciferase assays (*p* < 0.01, Figure 6(g-h)) were designed according to the binding sites of miR-20b-5p with MEG3 or CREB1 (figure 6(f)), and the obtained findings confirmed the presence of binding relationships between MEG3 and miR-20b-5p, and miR-20b-5p and CREB1. Moreover, we observed that miR-20b-5p was highly-expressed and CREB1 was poorly-expressed in the *in vitro* and *in vivo* models of UC, while M2-EV treatment reversed their expression patterns (*p* < 0.01, Figure 6(i-j)) and MEG3 over-expression could enhance the effect of M2-EVs (*p* < 0.01, Figure 6(j)). Collectively, these findings suggested that M2-EVs carried MEG3 into YAMC cells to competitively bind to miR-20b-5p, thereby promoting CREB1 transcription.

### miR-20b-5p over-expression attenuated the protective effect of M2-EVs on UC cells by reducing CREB1 transcription

**Figure 7.**
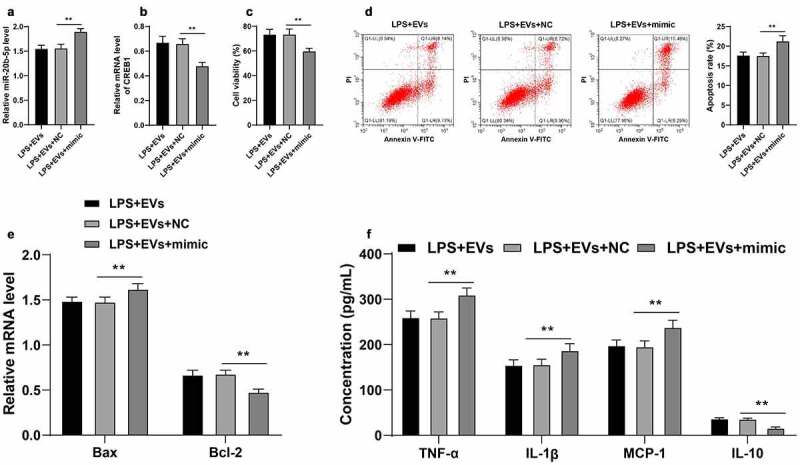
miR-20b-5p overexpression attenuated the protective effect of M2-EVs on UC cells by reducing CREB1 transcription. miR-20b-5p mimic was transfected into M2-EVs-treated YAMC cells, with mimic NC as control. a-b: miR-20b-5p and CREB1 expression in cells were detected using RT-qPCR. c-d: Cell viability and apoptosis were measured using CCK-8 assay (c) and flow cytometry (d). e: Bax and Bcl-2 mRNA levels were determined using RT-qPCR. f: The contents of inflammatory cytokines (TNF-α, IL-1β, MCP-1, and IL-10) were determined using ELISA. The experiment was repeated 3 times independently. Data are presented as mean ± standard deviation. Data in panels A-D were analyzed using one-way ANOVA, and data in panels E/F were analyzed using two-way ANOVA, followed by Tukey’s multiple comparisons test, **p < 0.01

Thereafter, the effects of miR-20b-5p and CREB1 on UC inflammation were verified. Briefly, miR-20b-5p mimic was transfected into M2-EVs-treated YAMC cells, which successfully augmented the miR-20b-5p expression (*p* < 0.01, Figure 7(a)) and down-regulated CREB1 mRNA levels (*p* < 0.01, Figure 7(b)). It was observed that up-regulation of miR-20b-5p significantly reduced the cell viability (*p* < 0.01, Figure 7(c)), promoted cell apoptosis (*p* < 0.01, Figure 7(d-e)), and enhanced inflammation (*p* < 0.01, figure 7(f)). Together, these findings indicated that miR-20b-5p over-expression attenuated the protective effect of M2-EVs on UC cells by reducing CREB1 transcription.

### miR-20b-5p over-expression attenuated the protective effect of M2-EVs on UC mice by reducing CREB1 transcription

**Figure 8.**
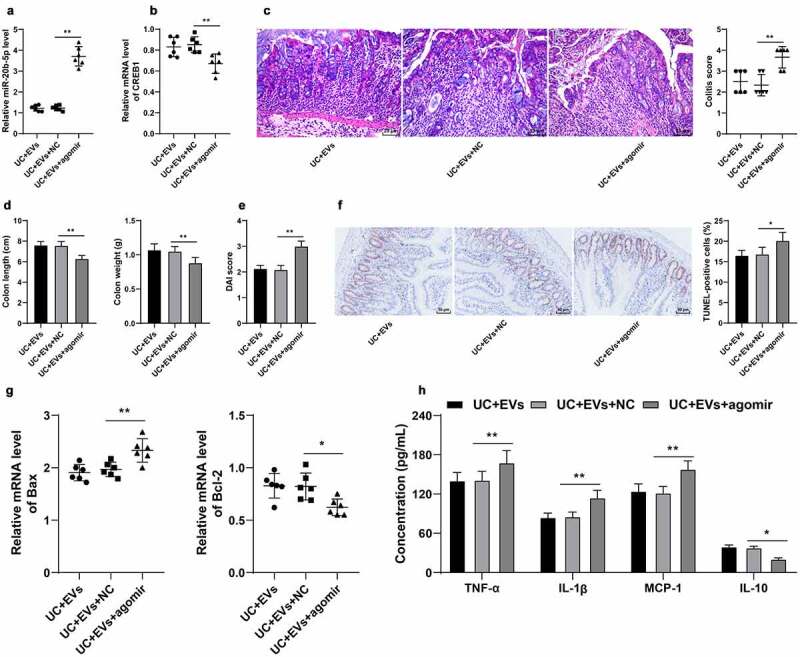
miR-20b-5p overexpression attenuated the protective effect of M2-EVs on UC mice by reducing CREB1 transcription. miR-20b-5p agomir was injected into mice in the M2-EVs group via tail vein, with control agomir as negative control. The colon tissues were collected on the 11th day. a-b: miR-20b-5p and CREB1 expressions were determined using RT-qPCR. c: HE staining and colitis score. d: Colon length and weight of mice in each group. e: DAI score of mice in each group. f: Apoptosis rate detected using TUNEL staining. g: Apoptosis-related factors (Bax and Bcl-2) detected using RT-qPCR. h: Inflammatory cytokines (TNF-α, IL-1β, MCP-1, and IL-10) determined using ELISA. N = 6. The experiment was repeated 3 times independently. Data in panels D/E/F/H are presented as mean ± standard deviation. Data in panels A-G were analyzed using one-way ANOVA, and data in panel H were analyzed using two-way ANOVA, followed by Tukey’s multiple comparisons test, **p < 0.01

Lastly, we validated the effects of miR-20b-5p and CREB1 on UC inflammation *in vivo*. miR-20b-5p agomir was injected into the mice in the M2-EVs group *via* tail vein, which successfully up-regulated the miR-20b-5p expression in mice (*p* < 0.01, Figure 8(a)) and down-regulated CREB1 mRNA levels (*p* < 0.01, Figure 8(b)). The results of HE staining illustrated that up-regulation of miR-20b-5p significantly aggravated the colon mucosal injury in mice and elevated the DAI score (Figure 8(c)), shortened the length of colon, reduced the weight, increased the DAI score (Figure 8(d-e)), and enhanced the level of apoptosis (figure 8(f-g)). Furthermore, ELISA results demonstrated that up-regulation of miR-20b-5p elevated the contents of TNF-α, IL-1β, and MCP-1, but decreased the content of IL-10 (Figure 8(h)). Altogether, these findings suggested that miR-20b-5p over-expression attenuated the protective effect of M2-EVs on UC mice by reducing CREB1 transcription.

## Discussion

UC is an autoimmune, chronic inflammatory disorder that affects the digestive system [44]. Macrophages are regarded as critical components of the innate immune system that can regulate intestinal microenvironment homeostasis [45]. It has been reported that Fenretinide, a synthetic retinol derivative, can regulate macrophage polarization to protect against experimental colitis induced by dextran sulfate sodium [46]. In addition, a prior study came across the ability of loganin, an iridoid monoterpenoid, to inhibit macrophage M1 polarization to attenuate UC [47]. These findings have indicated the potential role of macrophages in UC. Accordingly, the current study directly investigated the protective effect of M2 macrophage-derived EVs on DSS-induced colitis and further explored its downstream molecular mechanism, which verified the protective effect of M2 macrophages on colitis more comprehensively. Our findings elucidated that M2 macrophage-derived EVs carrying lncRNA MEG3 could alleviate UC inflammation *in vivo* and *in vitro via* the miR-20b-5p/CREB1 axis.

M2 macrophages are primarily implicated in a plethora of anti-inflammatory responses, such that induction of the M2 phenotype can ameliorate inflammatory bowel diseases including UC and Crohn’s disease [48]. Unsurprisingly, akin to their parent cells, M2-EVs are also known to play extensive roles in the pathology of inflammatory bowel diseases [49], and further serve as robust mediators for improvement of inflammation [11,50]. Herein our study, we adopted IL-4 to induce M2 polarization of mouse peritoneal macrophages, and then isolated M2-EVs. Subsequently, DSS-induced UC mice were treated with the obtained M2-EVs. It is particularly noteworthy that M2-EVs could notably prolong the colon length, augment the colon weight, decrease the DAI score, and further reduce apoptosis in UC mice. Additionally, M2-EV treatment brought about a reduction in the contents of pro-inflammatory factors, while enhancing the content of anti-inflammatory factors. Furthermore, we validated the effect of M2-EVs on UC inflammation *in vitro*, wherein YAMC cells were treated with LPS to simulate the inflammatory environment of UC *in vitro*. Subsequent findings illustrated that M2-EVs enhanced the cell viability, reduced apoptosis, and alleviated the inflammatory response of LPS-induced YAMC cells, which adds to the validity of our initial results. Consistently, a prior study has reported that M2-EVs can alleviate intestinal inflammation in DSS-induced colitis by mediating the CCL1/CCR8 axis [49]. Altogether, these findings and evidence suggest that M2-EVs are capable of reducing UC inflammation.

Furthermore, we explored the specific mechanism of M2-EVs in alleviating UC inflammation. Functioning as critical messengers, macrophage-derived EVs possess the ability to deliver multiple bioactive molecules such as lncRNAs from macrophages to recipient cells, thereby regulating the biological functions of recipient cells [10]. Meanwhile, dysregulated lncRNA expressions have been previously associated with the pathogenesis of UC [51]. More specifically, a prior study has uncovered that M2-EVs can reduce inflammation and protect against encephalomyelitis in mice by carrying a lncRNA [20]. One such lncRNA, namely MEG3, is well-documented to confer anti-inflammatory roles in various conditions [52–54]. For instance, the research performed by Du *et al*. has illustrated that MEG3 can disrupt LPS-induced inflammatory injury in sepsis [15]. Additionally, our findings demonstrated that M2-EVs transferred MEG3 into YAMC cells to up-regulate the MEG3 expression. To further elucidate the role of MEG3 in UC inflammation, we transfected LV-MEG3 into M2 macrophages and then isolated M2-EVs. Subsequent analyses revealed that M2-EVs over-expressing MEG3 could enhance the cell viability, reduce apoptosis, and relieve the inflammatory response of LPS-induced YAMC cells. Consistently, over-expression of MEG3 has been shown to relieve the severity of the intestinal ulcer, mitigate mucosal epithelial cell damage, and further alleviate inflammatory cell infiltration in UC rats, which is much in accordance with our findings [16]. Together, these findings make it plausible to suggest that M2-EVs confer a protective role on UC by delivering MEG3 into YAMC cells.

Additionally, we determined the downstream mechanism of MEG3 in UC inflammation, and found that MEG3 was primarily localized in the cytoplasm. It’s noteworthy that cytoplasmic lncRNAs are often implicated in the pathogenesis of UC by virtue of the lncRNA-miRNA-mRNA network [22]. Accordingly, we predicted the downstream miRNAs of MEG3 using online databases and obtained the intersection, among which miR-20b-5p is deregulated in UC patients, and the existing study has indicated miR-20b-5p to function as a novel potential biomarker for UC [42]. Furthermore, the downstream mRNAs of miR-20b-5p were predicted, wherein our efforts were focused on CREB1. Inherently, CREB1 is a transcription factor of the basic leucine zipper family that exerts an anti-inflammatory role in colonic diseases, including UC [55,56]. Further experimentation also confirmed the presence of binding relationships between MEG3 and miR-20b-5p, and miR-20b-5p and CREB1. Besides, we documented that miR-20b-5p was highly-expressed and CREB1 was poorly-expressed in the *in vivo* and *in vitro* models of UC, while M2-EV treatment resulted in decreased miR-20b-5p expression and increased CREB1 mRNA levels. These findings collectively suggested that M2-EVs carried MEG3 into YAMC cells to competitively bind to miR-20b-5p, thereby promoting CREB1 transcription. Moreover, we performed a series of functional rescue experiments by transfecting miR-20b-5p mimic into M2-EV-treated YAMC cells to up-regulate the miR-20b-5p expression and down-regulate the CREB1 expression. Existing evidence further indicates that miR-20b-5p overexpression can enhance the apoptosis rate and promote the inflammatory factor levels of Caco2 cells, resulting in aggravation of the intestinal permeability dysfunction in septic rats [30]. Similarly, CREB1 inhibition has been illustrated to promote inflammatory response and apoptosis in LPS-induced UC [43]. Consistently, our findings elicited that miR-20b-5p over-expression enhanced inflammation and attenuated the protective effect of M2-EVs on UC cells by reducing CREB1 transcription.

## Conclusion

To sum up, our findings revealed that M2-EVs carrying MEG3 competitively-binds to miR-20b-5p in colon epithelial cells and then promotes CREB1 transcription, thereby enhancing cell viability and reducing inflammatory responses in UC. However, our study solely determined the role of the MEG3-miR-20b-5p-CREB1 network in UC, while other factors of M2-EVs and their downstream mechanisms need to be further explored. We shall continue to verify the other functions of M2-EVs, and determine the role and mechanism of other cargoes of M2-EVs in UC in our future endeavors.

## Highlights

1. M2-EVs reduce the inflammatory response of UC mice.
2. M2-EVs reduce LPS-induced cellular inflammation.
3. M2-EVs carry MEG3 into cells to up-regulate MEG3 and reduce inflammation.
4. M2-EV-carried MEG3 competitively binds to miR-20b-5p and then promotes CREB1.
5. miR-20b-5p overexpression attenuates the protective effect of M2-EVs on inflammatory response in UC both in vivo and in vitro.
